# Determining the nurses’ perception regarding the effectiveness of COVID-19 protocols implemented in Eastern Province: Saudi Arabia

**DOI:** 10.3389/fpubh.2023.1291261

**Published:** 2024-01-05

**Authors:** Afnan Aljaffary, Tahani Al Elaiwi, Noot AlOtaibi, Fatimah AlAnsari, Arwa Alumran, Khaled F. Salama

**Affiliations:** ^1^Health Information Management and Technology Department, College of Public Health, Imam Abdulrahman Bin Faisal University, Dammam, Saudi Arabia; ^2^Environmental Health Department, College of Public Health, Imam Abdulrahman Bin Faisal University, Dammam, Saudi Arabia

**Keywords:** nurses, perception, COVID-19, protocols, preventive measures, PPE, hand hygiene

## Abstract

**Background:**

The global impact of Coronavirus Disease 2019 (COVID-19) has been profound, affecting public health, the global economy, and overall human life. Past experiences with global pandemics underscored the significance of understanding the perception of HCWs and hospital staff in developing and implementing preventive measures. The World Health Organization (WHO) provided protocols to manage the spread of COVID-19 and assist healthcare workers and health systems globally in maintaining high-quality health services.

**Objective:**

This study aims to assess nurses’ perception, awareness, and compliance regarding the implementation of COVID-19 protocols and explore factors influencing their perception.

**Methodology:**

A quantitative cross-sectional survey-based study was conducted, distributing a constructed survey among nurses in the Eastern Province of Saudi Arabia.

**Results:**

Out of 141 participants, most adhered to protocols such as hand sanitization, social distancing, and proper personal protective equipment (PPE) usage. The predominant age group among respondents was 31 to 40 years (*n* = 71, 50%). A significant portion of participants reported holding a bachelor’s degree (*n* = 86, 61%), with only 14% possessing advanced degrees (*n* = 19). Nearly a third of the nurses in the study had accumulated 6 to 10 years of professional experience (*n* = 49, 34.8%). A noteworthy percentage of nurses were engaged in daily shifts exceeding 8 h (*n* = 98, 70%). Gender differences were observed, with females exhibiting a higher tendency to avoid shaking hands and social gatherings. Saudi nationals were more inclined to shake hands and engage in gatherings. Non-Saudi nurses and those aged between <25 to 40 years demonstrated proper donning/doffing practices. Nurses with over 6 years of experience avoided social gatherings, while those working >8 h adhered better to PPE usage, proper donning/doffing, and disposal of PPE in designated bins.

**Conclusion:**

Understanding COVID-19 protocols is crucial for tailoring interventions and ensuring effective compliance with COVID-19 preventive measures among nurses. More efforts should be made toward preparing the healthcare nursing to deal with the outbreak. Preparing healthcare nursing with the right knowledge, attitude, and precautionary practices during the COVID-19 outbreak is very essential to patient and public safety.

## Introduction

The global impact of Coronavirus Disease 2019 (COVID-19), stemming from severe acute respiratory syndrome coronavirus-2 (SARS-CoV-2), has left an indelible mark on the world economy, public health, and individuals’ quality of life. In a brief span, it has placed an unprecedented burden on the global healthcare industry, necessitating every healthcare worker (HCW) to be at the forefront in managing the disease (1). The response to previous global pandemics underscored the pivotal role of HCWs and hospital staff perception in shaping and implementing protocols to address health crises (2).

In response to the COVID-19 outbreak, international health organizations like the World Health Organization (WHO) issued protocols to guide the management of COVID-19, aiding health systems and HCWs worldwide in maintaining the delivery of high-quality health services (3). Additionally, governments around the world implemented national protocols to contain the spread and impact of the pandemic. The global management of COVID-19 highlighted the effectiveness of a combination of non-pharmaceutical interventions, including lockdowns, school closures, restrictions on social gatherings and international travel, and robust information campaigns (4, 5). However, despite their effectiveness in curbing infections, these foundational protocols and policies fell short of completely halting the virus’s spread and containing the disease (6).

The timing of protocol implementation was crucial, with evidence demonstrating that earlier implementation significantly influenced virus control (7). HCWs exhibited outstanding performance in executing preventive protocols while addressing the clinical demands of the pandemic. Notably, there was a significant increase in HCWs’ adherence to preventive measures such as hand hygiene and the proper use of personal protective equipment (PPE) (8, 9). However, the swift adoption of protocols brought about various challenges.

HCWs, lacking prior experience in handling such diseases, faced high levels of stress, mitigated to some extent by protocols such as “disinfection efforts and isolation measures” that facilitated their work and maintained focus (10). PPE and resource shortages emerged as primary concerns, particularly in developing countries where these shortages hindered the implementation of preventive protocols (11, 12). Poorly designed infrastructure, including overcrowded Emergency Rooms (ER), hindered hospitals from implementing preventive measures such as social distancing (13, 14). Inadequate training, especially for redeployed HCWs facing increased workloads or requiring proficiency in using PPE, added to the challenges of managing the pandemic (2, 9, 11).

The spread of the pandemic has created drastic challenges and changes in all aspects of life, especially in health professionals’ education. One of the most important challenges is the preparedness and willingness of health professional nursing to work in infectious disease outbreaks (13, 14).

Therefore, assessing knowledge, attitude, and practices of health professionals regarding any infectious outbreak has become a fundamental step to setting an effective plan related to their preparedness. The initial research on COVID-19 has demonstrated that during unexpected natural crises and infectious diseases, health-care professionals will make every effort to participate in the efforts to control the outbreak and reduce the complications, but the less consciousness of the risk of the infection.

Constant changes in suggested preventive guidelines and protocols further complicated matters, causing confusion among HCWs about which protocol to adopt and how to implement it, leading to potential errors in handling cases (2). Nurses in Madrid emphasized the importance of hospital management and leadership considering feedback from frontline HCWs during the COVID-19 pandemic (15). Effective communication emerged as a crucial factor in clarifying applied preventive measures, ensuring proper implementation, avoiding misconceptions, and supporting HCWs through this challenging period (1, 2, 15, 16).

Nationally, Saudi Arabia implemented extraordinary and stringent preventive measures to safeguard citizens, ensure well-being, and enhance awareness, influencing a strong commitment to applying preventive measures (17). Papers highlighted that HCWs in Saudi Arabia possessed sufficient knowledge and skills to manage the COVID-19 outbreak (18, 19). Effective communication, leadership coordination, proactive planning, HCWs training, skill development, and the implementation of strict policies contributed to enhancing HCWs’ attitudes toward controlling the pandemic (20).

While published papers worldwide shared experiences in managing the COVID-19 outbreak, shedding light on various protocols advised and implemented by national and international agencies, including WHO and local governments, there remains a gap in research focusing on the perception of healthcare workers regarding the implementation of COVID-19 protocols in hospitals, particularly in Saudi Arabia. This paper seeks to address this gap by determining nurses’ perception, awareness, and compliance regarding the implementation of COVID-19 protocols and exploring the factors influencing their perception.

## Methodology

### Research design

This is a quantitative, survey-based, cross-sectional study among nurses in the Eastern Province in Saudi Arabia. The survey used a validated survey developed by Agarwal et al. (21). The survey measures the nurses’ perception, awareness, and compliance regarding the COVID-19 protocols and define the barriers in implementing them at the hospital. Survey results were analyzed using Statistical Package for Social Sciences (SPSS) to test the association between nurses’ socio-demographics and their awareness and perception of COVID-19 protocols.

### Study setting

Healthcare organizations operated during the pandemic of COVID-19 in Saudi Arabia.

### Participants

The participants were nurses who worked during the pandemic of COVID-19 in hospitals located in Eastern Province, Saudi Arabia. The sample size was 240. We distributed the survey through public social media accounts, also public nurses’ WhatsApp groups. Due to the scope of the study, we specifically targeted the nurses who faced or contacted the patients during that time as they always have direct contact with the patients and therefore, might be more exposed to get the infection compared to other healthcare workers.

### Instruments

An online survey was constructed based on a published validated survey done by Agarwal et al. (21) to evaluate the implemented preventive measures against COVID-19 among healthcare workers in India. It has two sections starting with Section A, which assesses the awareness and compliance toward the preventive measures. In addition, Section B cover the barriers to implementing these measures. Both sections cover the following elements: “hand hygiene, social distancing, personal protective equipment (PPE), gadgets/fomites, lifestyle, and exposure.” The elements of interest in this study are hand hygiene, social distancing, and PPE, which are implemented as COVID-19 protocols in hospitals in Saudi Arabia.

### Ethics and limitations

The ethical approval was obtained from the Institutional Review Board of Imam Abdulrahman Bin Faisal University; IRB-PGS-2021-03-443. The main limitation of the study journey was its short period as the study was conducted in one semester, which consequence in a small sample size.

### Analysis

Statistical Package for the Social Sciences (SPSS) software analyzed numerical data. Descriptive analysis was performed to present the participants’ characteristics, their reported practices in implementing the COVID-19 preventive protocols (Section A), and their perceived barriers toward implementing them (Section B). Furthermore, due to the small sample size (22), bivariate analysis was done using Fisher’s Exact test to assess the association between the participants’ characteristics and their perception and attitudes regarding the implemented COVID-19 protocols. Lastly, the significance of the results were based on the value of *p* (value of *p* = <0.05).

## Results

Out of the 141 nurses who completed the survey, 118 were females (83.7%), almost half of the participants were Saudis (*n* = 73). The majority of respondents were between the age of 31 to 40 years (*n* = 71, 50%). Most of the participants indicated that they have a bachelor’s degree (*n* = 86, 61%), while only 14% had higher degrees (*n* = 19). Almost third of the nurses in the study had 6 to 10 years of experience (*n* = 49, 34.8%). A remarkable number of nurses worked more than 8 h a day (*n* = 98, 70%%).

### Section I: adherence to prevention practices against COVID-19 infections among healthcare workers

Half of the nurses in the study indicated that they rarely shook hands when encountering a colleague (*n* = 73); on the other hand, 22% of them always or mostly did (*n* = 31). Most nurses in the study adhered to sanitizing their hands after meeting patients or touching their surroundings (*n* = 120, 85%), compared to only 5% who occasionally or rarely did (*n* = 7). Additionally, more than two thirds of the nurses followed the appropriate steps when washing or sanitizing their hands (*n* = 112, 79%).

A considerable number of nurses kept at least 1 meter when communicating with their colleagues (*n* = 96, 68%). Similarly, when asked about meeting colleagues at work for lunch gatherings, almost half of the nurses in the study mentioned that they occasionally or rarely did (*n* = 68), while 35% always or mostly did (*n* = 49).

Most of the nursers in the study (more than 80%) followed the proper steps for donning and doffing the PPE as per the guidelines, wore adequate PPE during duty, wore masks inside the hospital premises, cover both their nose and mouth with a mask while wearing it, and indicated that they dispose of PPE in specified colored dustbins after use according to guidelines (Table 1). More than half of the nurses in the study mentioned that they changed their PPE and did not reuse them in a single shift (*n* = 70, 50%). More than third of the nurses in the study mentioned that they always or mostly carry their face shields/gowns/PPE to their duty room in the ward before completely doffing (*n* = 55, 39%).

**Table 1:** Frequency (%) of responses to the adherence questionnaire.

Domain	Questions	Always	Mostly	Commonly	Occasionally	Rarely
Hand hygiene	I shake hands while meeting colleagues.	21 (14.9)	10 (7.1)	16 (11.3)	21 (14.9)	73 (51.8)
	I sanitize my hands after contact with each patient and/or his/her surroundings.	104 (73.8)	16 (11.3)	14 (9.9)	4 (2.8)	3 (2.1)
	I properly follow the steps of washing/sanitizing hands.	97 (68.8)	15 (10.6)	13 (9.2)	11 (7.8)	5 (3.5)
Social distancing	I maintain at least 1 m distance with coworkers at the hospital.	66 (46.8)	30 (21.3)	24 (17)	10 (7.1)	11 (7.8)
	I have attended social gatherings (meetings, lunch gatherings, visiting other coworkers’ offices, etc.) in the past two months.	27 (19.1)	22 (15.6)	24 (17.0)	29 (20.6)	39 (27.7)
Personal protective equipment (PPE)	I follow the steps of donning and doffing properly.	89 (63.1)	30 (21.3)	15 (10.6)	6 (4.3)	1 (0.7)
	I wear adequate PPE during duty (according to guidelines for my ward and patients).	103 (73.0)	17 (12.1)	8 (5.7)	10 (7.1)	3 (2.1)
	I wear a mask inside hospital premises.	106 (75.2)	12 (8.5)	10 (7.1)	8 (5.7)	5 (3.5)
	I cover both nose and mouth with a mask while wearing it	109 (77.3)	15 (10.6)	5 (3.5)	9 (6.4)	3 (2.1)
	I reuse my gowns/PPE during my single-duty shift.	28 (19.9)	13 (9.2)	14 (9.9)	16 (11.3)	70 (49.6)
	I carry face shields/gowns/PPE to my duty room in the ward before completely doffing.	30 (21.3)	25 (17.7)	19 (13.5)	15 (10.6)	52 (36.9)
	I dispose of PPE in specified colored dustbins after use according to guidelines.	93 (66)	22 (15.6)	18 (12.8)	2 (1.4)	6 (4.3)

Several variables are significantly associated with the participants adherence to Hand Hygiene. Saudis have significantly higher average adherence score to hand hygiene regulations compared to non-Saudis (*t* = 2.54, *p* = 0.012). Younger participants have significantly better hand hygiene adherence score compared to older participants (*f* = 3.085, *p* = 0.049). Further, participants with less than 6 working hours are more adherent to hand hygiene compared to more than 8 working hours (*t* = 2.76, *p* = 0.006, Table 2).

**Table 2:** Association between the participants’ characteristics and their perception toward the implemented COVID-19 protocols.

Variables	Freq. *n* = 141 (%)	Nurses’ adherence					
		Hand hygiene		Social distancing		Personal protective equipment (PPE)	
		Mean (SD)	Test (*p*-value)	Mean (SD)	Test (*p*-value)	Mean (SD)	Test (*p*-value)
Gender							
Male	23 (16.3)	6.2 (2.2)	*t* = 1.74 (0.084)	5.5 (1.5)	*t* = 2.021 (0.050)	5.5 (1.5)	*t* = 2.021 (0.050)Alth
Female	118 (83.7)	5.2 (2.6)		4.7 (2.1)		4.7 (2.1)	
Nationality							
Saudi	73 (51.8)	5.8 (2.3)	**t = 2.54 (0.012)**	5.4 (1.8)	**t = 3.72 (<0.001)**	5.4 (1.8)	**t = 3.720 (<0.001)**
Non-Saudi	68 (48.2)	4.8 (2.7)		4.0 (2.0)		4.2 (2.0)	
Age							
< 25–30	40 (28.4)	6.0 (2.7)	**f = 3.085 (0.049)**	5.1 (2.0)	*f* = 1.605 (0.205)	14.5 (5.4)	**f = 3.227 (0.043)**
31–40	71 (50.4)	5.3 (2.7)		4.9 (2.1)		12.0 (5.3)	
41 and above	30 (21.3)	4.5 (1.9)		4.3 (1.9)		12.8 (3.9)	
Education level							
Diploma	36 (25.5)	5.8 (2.5)	*f* = 0.1.168 (0.314)	5.1 (1.8)	*f* = 0.304 (0.738)	13.6 (4.8)	*f* = 0.503 (0.606)
Bachelor	86 (61.0)	5.1 (2.7)		4.8 (2.1)		12.6 (5.3)	
Higher education (MSc/PhD)	19 (13.5)	5.5 (2.1)		4.8 (2.0)		12.7 (5.1)	
Years of experience							
< 1–5 years	47 (33.3)	5.5 (2.4)	*f* = 0.574 (0.565)	5.4 (1.8)	*f* = 3.018 (0.052)	13.3 (4.4)	*f* = 0.250 (0.779)
6–10 years	49 (34.8)	5.5 (2.9)		4.5 (2.2)		12.6 (6.1)	
11 years or more	45 (31.9)	5.0 (2.3)		4.7 (1.9)		12.7 (4.7)	
Working hours							
< 8 h	43 (30.5)	6.2 (2.5)		5.3 (1.7)		5.3 (1.7)	
> 8 h	98 (69.5)	5.0 (2.5)	**t = 2.76 (0.006)**	4.7 (2.1)	*t* = 1.733 (0.086)	4.7 (2.1)	*t* = 1.73 (0.330)

Bold font indicates statistically significant results.

None of the variables in the study influence the adherence to social distancing except the nationality. The results show that Saudis are significantly more adherent to social distancing compared to non-Saudis (*t* = 3.72, *p* = <0.001, Table 2).

In addition, Saudi participants in the study appear to be significantly more adherent to Personal Protective Equipment compared to non-Saudis (*t* = 3.720, *p* = <0.001), and younger participants show significantly better adherence to PPE compared to older ones (*f* = 3.227, *p* = 0.043, Table 2).

### Section II: reasons for adherence or non-adherence to preventive practices from COVID-19 infection among healthcare workers

In this section, most nurses chose “not applicable,” which indicates that they did not face any difficulty or had any reason for not adhering to the applied COVID-19 protocols. Thus, the primary reported barriers/reasons are presented below.

#### Hand hygiene

Out of the 141 nurses, 17.7% were unaware that COVID-19 spread through handshaking, and 11.3% were not convinced that it does. Others felt it was inappropriate to refuse to shake another’s hand (17.7%), and some had difficulty changing their habits (12.8%). Moreover, 25.6% of respondents either were tired of continuously sanitizing their hands or did not have time due to their workload, whereas 10.6% faced a lack of sanitizers at their organizations. About following the sanitizing/handwashing steps, 17.7% did not find it crucial, 13.5% felt it was exhausting, and 12.1% did not have to follow all the steps (Table 3).

**Table 3:** Reasons for preventive practices among healthcare workers.

What is/are the reason(s) for not being able to don and doff properly?	
I do not know/remember the steps.	10 (7.1)
I believe they do not matter.	15 (10.6)
There is no dedicated doffing area.	23 (16.3)
There is a lack of helping people/mirrors to help in doffing.	15 (11.3)
There is a lack of sanitizers.	8 (5.7)
What is/are the reason(s) for not wearing adequate PPE?	
I do not know about guidelines for wearing PPE.	11 (7.8)
I do not believe adequate PPE protects me against COVID-19.	12 (8.5)
There are no guidelines for wearing PPE.	10 (7.1)
There is a lack of availability of PPE.	15 (10.6)
I find it uncomfortable to wear.	18 (12.8)
My long duty hours prevent its use.	9 (6.4)
I do not get time to wear PPE.	3 (2.1)
What is/are the reason(s) for not wearing a mask on the hospital premises?	
I do not know if masks are protective against COVID.	6 (4.3)
I do not find it useful.	8 (5.7)
I do not require it as I work in the administrative section.	7 (5.0)
There is a lack of availability.	9 (6.4)
I do not feel comfortable wearing it.	9 (6.4)
I have difficulty breathing while wearing it.	18 (12.8)
It gets hot while wearing them.	13 (9.2)
I do not look good wearing it.	1 (0.7)
Other reasons (Kindly specify):	1 (0.7)
What is/are the reason(s) for not covering both nose and mouth while wearing masks?	
I do not know if both nose and mouth have to be covered.	9 (6.4)
I do not find it useful.	9 (6.4)
I have difficulty breathing while wearing it.	31 (22.0)
I do not feel comfortable wearing it.	13 (9.2)
It slides down due to a loose fit.	11 (7.8)
Other reasons (Kindly specify):	1 (0.7)
What is/are the reason(s) for not disposing of masks properly in waste-bin/separate bags?	
I do not know if it should be kept properly in a waste bin/ separate bag.	19 (13.5)
I do not feel it is important to keep it properly.	9 (6.4)
I get too tired after work.	13 (9.2)
I do not find a suitable place to dispose of the mask.	27 (19.1)
What is/are the reason(s) for reusing gowns/PPE during a single shift?	
I do not know that I should not reuse the same gown/PPE.	13 (9.2)
I believe reusing them is not harmful.	20 (14.2)
There is a lack of availability of gowns/PPE.	28 (19.9)
I have long duty hours.	17 (12.1)
What is/are the reason(s) for not disposing of PPE in specific bins?	
I do not know how to dispose of PPE.	9 (6.4)
I believe proper disposing of PPE does not matter.	8 (5.7)
There is a lack of doffing area/dustbins.	14 (9.9)
I get confused regarding which PPE to dispose of in which bin.	35 (24.8)
I get too tired after duty hours.	8 (5.7)

#### Social distancing

14.2% of the nurses pointed out that lack of space hindered their ability to apply social distancing in hospitals and public places, and 17.0% found it hard to speak to others in public places. Additionally, 14.2% found it difficult to change their habits in the hospital, and equally, 14.2% did not see the necessity to keep a 1-meter distance for they wear their PPE all the time (Table 3).

#### Personal protective equipment (PPE)

Reasons for not wearing all the required PPE varied, starting with unavailability of PPE (10.6%), nurses feeling uncomfortable while wearing them (12.8%), or being unaware of the PPE guidelines (7.8%), and 8.5% were not convinced that the required PPE safeguard against COVID-19. Regarding wearing masks, 12.8% could not breathe easily, specifically when covering their nose and mouth (22.0%), 9.2% felt hot while wearing it, and 7.8% reported it sliding down from their nose. Moreover, nurses reused PPE due to its shortage in their organizations (19.9%) and long shifts (12.1%), whereas 14.2% did not see any risk in doing so (Table 3).

16.3% of nurses reported the unavailability of a designated area to doff, in addition to the need for an assistant or mirror to ensure proper doffing (11.3%) as barriers to applying the appropriate steps, and only 10.6% found it unnecessary to follow the steps of donning and doffing. When asked about PPE disposal, fatigue led 17.8% of nurses to not dispose of PPE/masks in their appropriate bins. Besides, 29.0% of the nurses pointed out the lack of designated bins. However, 13.5% were unaware that masks should be disposed of separately, and similarly, 24.8% were confused about which bin they should throw the PPE in Table 3.

Eighty-three percent of the respondents were female, in line with other studies (23) which indicate that 90% of the workforce during this COVID-19 crisis was female.

In the present study 69.5 of nurses spend longer working hours (> 8 h) might affect the efficiency and effectiveness of the workforce in delivering high-quality, safe care. A recent study in China about healthcare providers working longer hours due to the spread of COVID-19 conveyed high symptom rates of depression, insomnia, and work stress (24). An international study reported that when nurses wear personal protective equipment (PPE), they usually take 4–6 working hours without a break. This is very critical to nurses’ well-being, since longer hours wearing PPE can cause fatigue, stress, and exhaustion, making healthcare providers prone to causing medical errors (25). Hence, nursing administration should organize staffing and scheduling to avoid mental and physical health impairment.

Interestingly, in this study, demographics and work-related issues mattered. Female nurses had better preventive behaviors than male nurses as shaking hands with their colleagues. This distinction can be attributed to the fact that, in Saudi tradition and culture, females are more inclined to be healthcare providers than men (26). This result is consistent with a United Nations policy brief that women are more confident and have higher self-awareness about the impact of COVID-19 on women. Caution should be taken in interpreting this study, since only 16.3% of participants were male nurses, which means the findings cannot be generalized.

## Discussion

In this study, we found that, nationality, age, and working hours influence nurses’ different perceptions regarding the effectiveness of COVID-19 protocols. Based on the perception of the nurses regarding the COVID-19 protocol, we found that 79.4% of the respondents followed the appropriate steps in washing their hands, and 85.1% wore their PPE according to their guidelines as compared with Social Distancing wherein only 16.1% of the participants keep at least a meter when communicating. This means that participants perceive both hand washing and wearing PPE as effective protocols against the pandemic. Previous studies have concluded that nurses have demonstrated outstanding performance in conducting preventive protocols to meet the demands of the pandemic. Notably, nurses increasingly adhere to preventive measures such as hand hygiene and wearing PPE (8, 9).

Furthermore, the study reveals that more than 10 % of the participants are not wearing all the required PPE because of its unavailability. Previous researchers also noted that the shortage in PPE and resources is a major concern for healthcare workers (11, 12). In other words, one of the main reasons nurses are not wearing their PPE despite having a positive perception of the protocol is because there is a shortage of this resource.

The earliness of implementation determines the effectiveness of COVID-19 protocols implemented in 2020 to 2021 in response to the pandemic. We found that nurses trust PPE use as an effective COVID-19 protocol; once they wear it, they do not see the need to keep a 1-meter distance or have social distancing anymore. Researchers have proven that the earlier the protocols are implemented, the more remarkable the impact (27). In addition, COVID-19 protocols are more effective by combining non-pharmaceutical interventions such as lockdowns, restricting social gatherings and international travels, school closures, and strengthening information campaigns. As for healthcare workers, previous studies noted that effective communication between HCWs, patients, leadership, and team coordination and implementing strict policies to avoid errors and control the pandemic are effective COVID-19 protocols (21).

After comparing the social demographic with the nurse’s perception of COVID-19 protocol, the education factor was found not to influence nurses’ different perceptions regarding the effectiveness of COVID-19 protocols. This finding is consistent with Olum et al. (28) study, which revealed that there is no association between level of education and compliance with COVID-19 protocols. This can be justified by the fact that the level of knowledge about COVID19 precautions the level of knowledge about COVID-19 might be similar irrespective of level of education of healthcare workers (28).

Although the gender is not significantly associated with social distance practice in the present study females are more cautious about shaking hands with their colleagues, which means that they are more likely to social distance than their male counterparts. This can be supported by another study which revealed that female nurses had significantly higher good hand hygiene practice than male nurses (24). It was justified that the higher compliance rate of hand hygiene among females may also be associated with their propensity to practice socially acceptable behaviors (25).

Moreover, non-Saudi nurses had more tendencies to shake hands and attend gatherings with their colleagues than Saudi nurses. Therefore, non-Saudi nurses are more likely to not adhere to social distancing protocols than Saudi nurses. In addition, Saudi nurses are more likely to follow the proper steps in hand hygiene compared with non-Saudis.

Nurses who are 25 to 40 years of age avoid entering their duty rooms with their face shields and are more likely to perform appropriately donning than nurses 40 years of age and above and Nurses who were 31 years and above are less likely to reuse their PPE for a single shift. Moreover, younger participants have significantly better hand hygiene adherence scores compared to older participants. We also found that the more experienced the nurses are, the more they comply with the COVID-19 protocols. This finding is supported by another study that has been conducted in Nigeria which revealed that compliance with the preventive measure significantly increased as nurses’ years of experience increased (29).

We also found that compared to nurses who worked more than 8 h, those who worked for less than 8 h adhered more to hand hygiene. This shows that nurses who are overburdened are less likely practicing proper handwashing. Previous studies showed that not having previous experience handling certain diseases impacted the HCWs’ perception and behavior on COVID-19 protocols; thus, experience influences how nurses handle the pandemic (29).

In the present study, the overall compliance with PPE usage and IPC measures in the nurses was 85.1%. However, the discrepancy in compliance rates reported in different studies might be attributed to the time factor. Some studies were conducted during the first wave of the COVID-19 pandemic (30, 31).

High perception, good level of knowledge, and high compliance rate reported among nurses in this study. Similar findings were reported in Abdel Wahed et al. study conducted among HCWs in Egypt (32).

Nurses in this study reported higher preventive practices in dealing with COVID-19. These findings affirm a previous study among healthcare workers in Saudi, and a recent study about COVID-19 in India among students and health care workers, in which, due to constant exposure and previous outbreak experience with similar coronavirus disease, this nurses were able to practice in their full clinical capacity and use preventive measures (25).

This study revealed that good compliance with PPE usage, hand hygiene, and IPC measures was independently predicted by nurses’ risk perception and knowledge about PPE usage and hand hygiene. Likewise, Brooks et al. (33) review studied 56 papers and revealed evidence that staff with higher concern about the risk of infection were more likely to comply with the recommended measures. Similarly, Webster et al. (34) review found that accurate knowledge about the recommended performances, perception of susceptibility and severity of being infected, and perception of benefits of compliance would facilitate compliance.

The nurses did not see “availability of incorrect PPE sizes, feeling uncomfortable and irritable when wearing PPE, as factors that influence their practice of preventive measures against COVID-19. It is vital to the importance to apply preventive measures and comply with PPE usage, hand hygiene, and IPC measures that could minimize the spread of the disease (35). Despite that negative influence of PPE on nurses and some other psychological factors were reported by Chan (36) in their study, which is not the case in the present study, there is a need for improved knowledge through sufficient training in order to enhance compliance to the preventive measures to COVID-19 and stop all the improper practices that may spread the infection.

In accordance with the present study, the study of Al-Rawajfah et al. (37), revealed that the overall knowledge of the health-care students about the current COVID-19 is not optimal, as only about one-quarter of the sample scored more than 75% of the maximum score.

Variation in compliance rates reported in studies could also be explained by the disparity in the studies’ methodology. Self-reporting might overestimate the real compliance rate unlike assessing an observed practice. Similar results revealed from the study of Al-Mugheed et al. (26) who investigated the acceptance and attitudes of nursing students toward the COVID-19 vaccine booster dose in two Gulf Cooperation countries and showed that the total attitude scores for the students ranged from 28 to 35, with a mean score of 15.8 (SD = 2.5), representing 73% of the highest possible score, with 79.3% classified as ‘positive attitude toward booster dose of COVID-19′ as vaccine booster might cause infection, vaccine booster ineffective, worried about adverse effects and not safe were major barriers influencing the acceptance of the COVID-19 vaccine booster. However, preparing nursing students with positive attitude of COVID-19 vaccine booster is very important to patient and community safety.

## Conclusion

The purpose of the current research is to define the nurses’ perception, awareness, and compliance regarding COVID-19 protocols implementation and explore the factors influencing their perception. Through a quantitative, survey-based, cross-sectional study, we identified the nurse’s perception regarding COVID-19 protocol, determined the effectiveness of COVID-19 protocols implemented from 2020 to 2021, and compared nurses’ social demographic with their perception toward COVID-19 protocols. As a result, we found out that nurses perceive hand hygiene and wearing of PPE as effective COVID-19 protocols and that, nationality, age, and working hours influence nurses’ different perceptions regarding the effectiveness of COVID-19 protocols.

These findings suggest that different social demographic factors influence how nurses perceive COVID-19 protocols. Healthcare providers should consider these differences in training nurses and healthcare workers in adhering to COVID-19 protocols. For example, since non-Saudis are less likely to social distance than Saudis, more informative training should be given to non-Saudi nurses regarding the importance of social distancing. Since nurses who are working for more than 8 h a day are less likely to follow the protocols, they should be given more training and their perceptions should be considered while implementing COVID-19 protocols in hospitals and healthcare centers, in order to assure better adherence in their busy schedule.

The project’s strong points include filling in the research gap on the perception of healthcare workers regarding the implementation of the COVID-19 protocol in hospitals, especially in Saudi Arabia. Moreover, we found social demographics that affect nurses’ perceptions of the protocols. However, the paper was based on a structured survey; thus, presenting a limitation in the study. Future researchers can conduct interviews to confirm the study’s findings and find a more in-depth explanation of why nurses provided the answers they gave. Indeed, further study is required to understand nurses’ perceptions regarding the effectiveness of COVID-19 Protocols implemented in Saudi Arabia.

## Data availability statement

The original contributions presented in the study are included in the article/supplementary material, further inquiries can be directed to the corresponding author.

## Ethics statement

The participants provided written informed consent to participate in this study. The ethical approval was obtained from the Institutional Review Board of Imam Abdulrahman BinFaisal University; IRB-PGS-2021-03-443.

## Author contributions

AfA: Formal analysis, Investigation, Methodology, Supervision, Validation, Writing – original draft, Conceptualization, Data curation. TA: Investigation, Methodology, Validation, Writing – original draft, Visualization, Writing – review & editing. NA: Investigation, Methodology, Validation, Writing – original draft, Writing – review & editing, Conceptualization, Data curation. FA: Data curation, Investigation, Methodology, Validation, Writing – original draft, Writing – review & editing. ArA: Data curation, Formal analysis, Investigation, Methodology, Supervision, Validation, Writing – original draft, Writing – review & editing. KS: Data curation, Formal analysis, Investigation, Methodology, Supervision, Validation, Writing – original draft, Writing – review & editing, Visualization.
